# Factors Influencing Preparedness for Bioterrorism among Koreans

**DOI:** 10.3390/ijerph18105401

**Published:** 2021-05-18

**Authors:** Yujeong Kim, Eunmi Lee

**Affiliations:** 1 Research Institute of Nursing Science, College of Nursing, Kyungpook National University, Daegu 41944, Korea; yujeongkim@knu.ac.kr; 2 Department of Nursing, Research Institute for Basic Science, Hoseo University, Asan 31499, Korea

**Keywords:** bioterrorism, biological warfare, emergency preparedness, coping behaviors

## Abstract

Bioterrorism is destructive enough to cause a societal collapse, and preparation for bioterrorism is imperative. This study aims to identify the factors influencing preparedness for bioterrorism among Koreans. A total of 1050 subjects were included in the study, which were allocated according to region and age in proportion to population. An online survey was used to examine the following factors: participants’ general characteristics; cognitive factors including perceived probability, perceived seriousness, perceived personal impact, perceived coping efficacy, and perceived resilience; social–contextual factors including perceived governmental preparedness and perceived front-line preparedness; affective responses including affective response to terrorism and anxiety; and bioterrorism preparedness. The factors influencing the level of preparedness for bioterrorism included age, marital status, experience of bioterrorism education, perceived personal impact, perceived coping efficacy, perceived resilience, and perceived front-line preparedness. The factors that most significantly affected the level of preparedness for bioterrorism were perceived coping efficacy and perceived front-line preparedness, with an R^2^ of 41.4%. Relevant education and public relations programs should be strengthened to help citizens minimize their exposure and known to inform relevant institutions in the event of suspected bioterrorism, and front-line responders should cultivate their ability to respond to bioterrorism quickly and accurately.

## 1. Introduction

Bioterrorism refers to the deliberate use of biological agents such as viruses, bacteria, toxins, or fungi with the intention to cause chaos in society [1,2]. Such intentional acts have been part of the history of war and terrorism, and since the 2001 anthrax attacks in the United States, the importance of actively preparing for potential bioterrorism events has been recognized. Biological agents (e.g., anthrax, smallpox, botulinum toxin, plague, viral hemorrhagic fevers, and tularemia) can easily spread and can cause disruption in public health systems and society [1]. Bioterrorism agents are easy to produce, conceal, transport, and release, difficult to detect early due to incubation periods, and marked by infection characteristics such as long-term prevalence and high mortality rate [3]. Global terrorism is a rapidly growing threat to global security that also increases the risk of bioterrorism [4]. Therefore, an efficient medical response system should be in place to minimize potential damage, and continuous efforts to strengthen the national medical response system are extremely important [3,5].

The ongoing COVID-19 pandemic has shown how toxic organisms can disrupt human life and public health systems and can put the world at great risk. In addition, it has revealed limitations of medical advancement and medical response systems. Without excellent understanding and the ability of the public to respond to bioterrorism, the medical response system could easily collapse. In particular, although South Korea is striving for peace, its situation as a divided country with North Korea means that the possibility of bioterrorism in South Korea cannot be completely eliminated [6]. Therefore, South Korea should not only conduct military training [7] but also ensure that its citizens are equipped with the ability to respond effectively to bioterrorism.

Lee and Lemyre (2009) suggested a social–cognitive model of individual response to terrorism in relation to chemical, biological, radiological, nuclear, and explosive (CBRNE) terrorism [8]. This model explains that the following factors influence an individual’s preparedness for bioterrorism: cognitive factors, such as risks of terrorism; social–contextual factors, such as the government and front-line response; and affective responses, such as concerns about terrorism.

The protection motivation theory [9] recognizes cognitive factors such as the degree to which an individual perceives a particular risk as a probable and a serious threat, and the degree to which they perceive themselves as being able to cope with it, as key determinants of preventive behavior. According to previous studies, perceived probability of the occurrence of terrorism in one’s own country, perceived seriousness of the consequences of terrorism, perceived personal impact of terrorism, and perceived coping efficacy are related to an individual’s preparedness for terrorism [8,10]. In addition, perceived resilience, which refers to an awareness of one’s ability to cope with the stress caused by extreme crisis situations, such as terrorism, can also have an impact on terrorism preparedness [11].

In Paton’s social–cognitive model of disaster preparedness, individuals’ trust in authorities to regulate disaster and affective response to a disaster, such as anxiety and worry, induce motivation for disaster preparedness [12]. Concerning social–contextual factors, Lee and Lemyre (2009) established that perceived governmental preparedness and credibility of front-line workers (first responders, hospital and health care service providers, and nongovernmental and local community organization staff) are related to individual bioterrorism preparedness [8]. Regarding affective responses, emotional responses such as worrying about biological terrorism and the level of individual anxiety can affect the motivation to prepare for disasters such as terrorism [8,12]. In addition, Slovic and Weber (2002) reported that demographic factors also affect individuals’ recognition and preparedness for terrorism [13].

Although South Korea has various bioterrorism risk factors, there are almost no studies that examine Korean individuals’ preparedness for bioterrorism. Some studies on bioterrorism preparedness of military nursing officers and nursing students have been conducted [14,15], including a study on the development of citizen response behavior during special disasters such as bioterrorism [16]. However, research on the bioterrorism preparedness of the general public is still scarce.

Therefore, this study aimed to identify the factors influencing individuals’ preparedness for bioterrorism. It was hypothesized that individuals’ preparedness for bioterrorism would have a significant relationship with cognitive factors (perceived probability, perceived seriousness, perceived personal impact, perceived coping efficacy, and perceived resilience), social–contextual factors (perceived governmental preparedness and perceived front-line preparedness), and affective responses (affective response to terrorism and anxiety).

## 2. Materials and Methods

### 2.1. Design

This study was a cross-sectional survey designed to identify factors affecting the level of preparedness for bioterrorism among Koreans.

### 2.2. Study Population

This study targeted people between the ages of 18 and 65 residing in Korea. Participants were gender-equally extracted from the 17 administrative divisions of South Korea, allocated according to region and age in proportion to population. The data were collected via online surveys from 24 September to 29 September 2020. The participants were recruited by a research institution specializing in large-scale surveys. Using G*Power 3.1 (Faranz Faul, Universität Kiel, Germany), the effect size was set at 0.02, the power was set at 0.95, α was set at 0.05, and the number of predictors was set at 5, indicating a minimum number of participants of 995. Considering the dropout rates for online surveys, the link to the survey was sent to 2537 people, and a total of 1050 completed the survey and were included in this study. Those who clicked the link to the online survey read the disclaimer about the purpose and method of the study and agreed to the survey content before beginning the survey. The survey took approximately 15 min.

### 2.3. Measures

#### 2.3.1. General Characteristics

Questions about the following general characteristics were included: age, gender, education level, marital status, children, occupation, financial status, health status, and experience of bioterrorism education. Education level was classified as middle school or less, high school, and university or higher. Marital status was divided into married and unmarried/divorced/separated. Occupation was classified into the following seven categories, according to the Korean standard job classification by Statistics Korea: managers, experts; clerks; sales and service workers; skilled agricultural and fishery workers; technical workers, plant and machine operators and assemblers; simple labor workers; or unemployed (housewife, student, etc.). Financial status and health status were classified as low, moderate, and high. The experience of bioterrorism education was assessed as yes or no.

#### 2.3.2. Cognitive Factors

The following cognitive factors were examined: perceived probability, perceived seriousness, perceived personal impact, perceived coping efficacy, and perceived resilience.

Perceived probability was measured with a tool developed by Rebmann and Mohr [10]. A five-point Likert scale ranging from (1) “not at all” to (5) “very much” was used to measure participants’ response to the following two items: “A bioterrorism attack is likely to occur somewhere in the Korea, during the next 5 years,” and “A bioterrorism attack is likely to occur in my community during the next 5 years.” Points from each question were added up, and a higher score indicated a higher awareness of the possibility of bioterrorism. In this study, the Cronbach’s α value was 0.911.

Perceived seriousness, perceived personal impact, and perceived coping efficacy were measured using questions from Lee and Lemyre’s CBRNE terrorism [8]. The following questions were used to examine each factor: “How serious do you think it would be if bioterrorism did occur in Korea?” was used to measure the perceived seriousness; “If bioterrorism occurred in Korea, to what extent do you think it would have an impact on your life?” was used to measure the perceived personal impact; and “If bioterrorism occurred in Korea, how well do you think you would be able to cope with it?” was used to measure the perceived coping efficacy. All three questions were rated on a five-point Likert scale ranging from (1) “not at all” to (5) “very much”. A higher score indicated higher perceived seriousness, perceived personal impact, and perceived coping efficacy.

Perceived resilience was measured using the Connor–Davidson Resilience Scale developed by Connor and Davidson [11]. This tool consists of a total of 25 questions measured using a five-point Likert scale ranging from (0) “not at all” to (4) “very much”. A higher score indicated higher perceived resilience. The reliability of this tool at the time of development was Cronbach’s α = 0.89, and the reliability in this study was Cronbach’s α = 0.932.

#### 2.3.3. Social–Contextual Factors

Social–contextual factors including perceived governmental preparedness and perceived front-line preparedness were measured with two questions using the tool from Lee and Lemyre [8]. Perceived governmental preparedness refers to the perception of preparedness of governmental institutions for bioterrorism. In the original study, governmental institutions were divided into three parts: the federal government, the provincial government, and the municipal government. In this study, the three sections were categorized as the federal government, provinces, and cities to reflect the characteristics of administrative division in Korea. A five-point Likert scale ranging from (1) “not at all” to (5) “very much” was used. A higher score indicated a stronger perception that governmental institutions are prepared for bioterrorism. The reliability of the tool in the original study was Cronbach’s α = 0.81, and the reliability in this study was Cronbach’s α = 0.940.

Perceived front-line preparedness refers to the perception of preparedness of institutions that play a front-line role in emergency situations such as bioterrorism. These include first responder organizations (e.g., police officers, paramedics, firefighters), hospital and health care services, nongovernmental organizations (e.g., the Red Cross, the Salvation Army), and local community organizations (e.g., religious organizations, social clubs). A total of four questions examined the preparedness of each group of front-line organizations for bioterrorism. A five-point Likert scale ranging from (1) “not at all” to (5) “very much” was used. A higher score indicated stronger perception that groups are well prepared for bioterrorism. The reliability of the tool in the original study was α = 0.77, and the reliability of this study was α = 0.857.

#### 2.3.4. Affective Responses

Affective responses were measured by evaluating affective response to terrorism and anxiety. Affective response to terrorism was measured by a question from Lee and Lemyre [8]: “To what extent do you currently worry about terrorism in Korea?” A five-point Likert scale ranging from (1) “not at all” to (5) “very much” was used. A higher score indicated higher concern about bioterrorism.

Anxiety was measured using the tool from the Beck Anxiety Inventory (BAI), which was developed by Beck et al. [17] and translated into Korean by Kwon and Oei [18]. It consists of 21 items about the feeling of anxiety during the last week, measured on a four-point Likert scale ranging from (0) “never” to (3) “very strongly”. The score ranged from 0 to 63, and a higher score indicated higher anxiety. In the Korean version, the results were categorized based on the sum of the points. For example, scores less than or equal to 21 were classified as “not anxious (normal)”; scores between 22 and 26 were classified as “anxious (requiring observation and intervention)”; scores between 27 and 31 were classified as “state of severe anxiety”; and scores greater than or equal to 32 were classified as “state of extreme anxiety”. In this study, the reliability of the tool was Cronbach’s α = 0.941.

#### 2.3.5. Bioterrorism Preparedness

Bioterrorism preparedness was measured using the tool developed by Kat et al. [19]. A total of four items consisted of questions about effective response to bioterrorism attacks, access to information about bioterrorism, phone calls in the event of suspected bioterrorism attacks, and how to identify bioterrorism substances. A five-point Likert scale ranging from (1) “not at all” to (5) “very much” was used. The scores from each item were added up, and a higher score indicated that the individual was more prepared for bioterrorism. The reliability of the tool in this study was α = 0.911.

### 2.4. Statistical Analysis

The collected data were analyzed using SPSS 22.0 (IBM Corp., Armonk, NY, USA). The participants’ general characteristics were analyzed for frequency, percentile, average, and standard deviation, using statistical analysis. The average, standard deviation, maximum value, and minimum value were calculated for the major variables. The participants’ level of preparedness for bioterrorism according to their general characteristics was measured using *t*-test, one-way analysis of variance (ANOVA), and the Scheffé test. The associations between the preparedness for bioterrorism and cognitive factors, social–contextual factors, and affective responses were analyzed using Pearson’s correlation. To identify the factors influencing the preparedness for bioterrorism, multiple linear regression was performed including the valid variables from the univariate analysis.

## 3. Results

### 3.1. Difference in Level of Preparedness for Bioterrorism by the Participants’ General Characteristics

The participants’ general characteristics are presented in Table 1. The number of participants in each age group was proportionate to the Korean population, and the average age was 42.46 years old. Most participants had an education level of university or higher, 62.9% of the participants were married, and 57.8% had children. The largest group was unemployed, including housewives and students, followed by office workers. More than half of the participants reported moderate financial and health status, and 79.5% of the participants had not received bioterrorism education.

The level of preparedness for bioterrorism was significantly higher among men than women, and the age group between 60 and 65 was significantly more prepared for bioterrorism than other age groups. The participants with the following characteristics were significantly more prepared for bioterrorism: single, with children, health status above moderate, and experienced bioterrorism education. Although there was a statistically significant difference in the level of preparedness for bioterrorism by financial status, this difference was not significant based on the post hoc test. There was no statistically significant difference in the level of preparedness for bioterrorism by other general characteristics.

### 3.2. Level of Major Variables

The scores for cognitive factors, social–contextual factors, affective responses, and bioterrorism preparedness are shown in Table 2.

### 3.3. Correlations among Major Variables

There were significant relationships between the preparedness for bioterrorism and perceived seriousness, perceived personal impact, perceived coping efficacy, and perceived resilience. Among social–contextual factors, there were significant relationships between the preparedness for bioterrorism and perceived governmental preparedness and perceived front-line preparedness. All social–contextual factors had a significant relationship with preparedness for bioterrorism. The relationship of items under perceived probability and affective responses were not statistically significant with preparedness for bioterrorism (Table 3).

### 3.4. Factors Influencing the Preparedness for Bioterrorism

To identify the factors influencing preparedness for bioterrorism, a stepwise multiple regression analysis was performed using the general characteristics that had a statistically significant relationship with preparedness for bioterrorism (including gender, age, marital status, children, financial status, health status, experience of bioterrorism education) and the factors that had a significant relationship with preparedness for bioterrorism (including perceived seriousness, perceived personal impact, perceived coping efficacy, perceived resilience, perceived governmental preparedness, and perceived front-line preparedness) as independent variables. To perform the regression analysis, the multicollinearity between autocorrelation and independent variables was verified. The Durbin–Watson value was 1.93, which is close to the standard value of 2, thus confirming that there is no autocorrelation. Tolerance was above 0.1, ranging from 0.76 to 0.96. The multicollinearity between independent variables was tested using variance inflation factors (VIF). The VIF was ≈1.04–1.32, which was lower than 10. Therefore, it was confirmed that there was no problem of multicollinearity.

The results of the regression analysis showed that the factors influencing the preparedness for bioterrorism are gender, marital status, experience of bioterrorism education, perceived personal impact, perceived coping efficacy, perceived resilience, and perceived front-line preparedness. Perceived coping efficacy and perceived front-line preparedness are the factors with the most significant influences. The R-squared value explaining the influences of all variables on the preparedness for bioterrorism was 41.4% (Table 4).

## 4. Discussion

The results showed that men and married participants were more prepared for bioterrorism. This result is similar to a previous study that reported that male and married individuals with children are more aware of or prepared in response to bioterrorism [20]. In addition, experience of bioterrorism education had a significant impact on the level of preparedness for bioterrorism. As shown in previous studies, continuous education on bioterrorism plays an extremely important role in responding to bioterrorism, and various studies are being conducted to determine effective educational content and methods [8,10,21]. In addition, the Korea Disease Control and Prevention explains information about bioterrorism preparedness and response information in detail on its website, and the government receives daily reports to monitor the level of risk of bioterrorism and infectious diseases through the bioterrorism and emergency room syndrome monitoring systems [22]. In addition, regulation of manufacturing and import/export restrictions on specific chemical substances and biological agents has been implemented since January 1, 2007 [23]. In particular, the prolonged COVID-19 pandemic increased public awareness of the dangers of biological substances and confirmed that continuous guidance, announcements, public relations, and education from the national disaster response system were effective ways to prevent the spread of infectious diseases [24]. Therefore, it is necessary to provide systematic and effective education on bioterrorism at a national level to cultivate the ability to respond to bioterrorism.

Among cognitive factors, the perceived personal impact, perceived coping efficacy, and perceived resilience affected the level of preparedness for bioterrorism. Previous studies also reported that all cognitive factors were associated with the preparedness for bioterrorism [8], and the lower the perceived coping efficacy, the less prepared for bioterrorism they were and the more concerns they had [8,25]. However, in some studies, when perceived personal impact was excessively high, the level of preparedness was rather reduced due to the feeling of helplessness toward the threat of bioterrorism [8]. Therefore, the level of preparedness for bioterrorism increases when individuals feel that they can control and overcome the event through perceived resilience [26,27,28,29].

Finally, among social–contextual factors, perceived front-line preparedness was most highly associated with preparedness for bioterrorism. This result extended findings from a previous study, which reported that individuals’ perceived coping efficacy was associated with perceived preparedness [26,28,30], and it is connected to a study that showed that perceived front-line preparedness among social–contextual factors improved individual preparedness and reduced avoidance behaviors, thus positively influencing preparedness for bioterrorism [8,31,32]. These results show that an individual’s level of preparedness increases in the following situations: when the dangers of bioterrorism are recognized; when the perceived front-line preparedness increases from the social–contextual perspective; and when individual coping efficacy improves.

This study has a few limitations. First, the survey was conducted anonymously and was a self-report study about individuals’ own awareness, attitudes, and capability. Thus, the responses could have been influenced by social desirability bias. Second, this study was a cross-sectional survey, and it is difficult to clearly identify the causation among variables. However, these results are expected to be used as basic information for directing future research and application programs, as the factors influencing the level of preparedness for bioterrorism have been identified. Third, the participants were limited to the age group between 18 and 65 to ensure the reliability of the online survey, and it is difficult to generalize the study results to all Koreans because the factors influencing the preparedness for bioterrorism among children, adolescents, and the elderly were not identified in this study. Moreover, since the online survey was conducted within a short period, the representative sample may not be attained. In the future, research through individual interviews should be conducted to improve these limitations. However, despite these limitations, the value of this study is that the important factors in bioterrorism preparedness of Korean people living in a divided country with a high terrorism risk are their own coping efficiency and confidence toward first responders. Based on the results of this study, it can be used to provide vital basic information necessary in developing programs (policy, public relations, education, and training) to improve the ability to respond to bioterrorism.

## 5. Conclusions

This study was performed to identify factors influencing preparedness for bioterrorism among Koreans. Perceived coping efficacy and perceived front-line preparedness had the most significant influence on the level of preparedness for bioterrorism. Therefore, relevant education and public relations programs should be strengthened to encourage citizens to minimize their exposure and inform relevant institutions in the event of suspected bioterrorism, and front-line responders should cultivate their ability to respond to bioterrorism quickly and accurately. To strengthen the level of constant preparedness for bioterrorism, understanding the people’s perception of the severity of bioterrorism, level of preparedness, and coping ability is important in facilitating rapid response to bioterrorism and equipping the ability to respond to national crisis situations. Therefore, in order to come up with various approaches based on the people’s awareness and understanding of preparation and coping ability, the efforts of experts and mediation based on this should be prepared.

## Figures and Tables

**Table 1:** Bioterrorism preparedness according to general characteristics of the subjects. (*N* = 1050).

Variables	Categories	*n* (%) M ± SD	Bioterrorism Preparedness	
			Mean ± SD	t or F(*p*) Scheffé
Gender	men	525 (50.0%)	9.15 ± 3.49	5.40 (<0.001)
	women	525 (50.0%)	8.01 ± 3.34	
Age	18–29 ^a^	227 (21.6%)	8.26 ± 3.37	3.73 (0.005) e > a,c
	30–39 ^b^	200 (19.1%)	8.49 ± 3.53	
	40–49 ^c^	242 (23.0%)	8.26 ± 3.43	
	50–59 ^d^	250 (23.8%)	8.76 ± 3.36	
	60–65 ^e^	131 (12.5%)	9.53 ± 3.60	
	total	42.46 ± 12.95	8.58 ± 3.46	
Education level	middle school or less	12 (1.2%)	8.58 ± 4.56	1.21 (0.299)
	high school	248 (23.6%)	8.28 ± 3.32	
	university or higher	790 (75.2%)	8.67 ± 3.48	
Marital status	no	390 (37.1%)	8.14 ± 3.32	−3.21 (0.001)
	yes	660 (62.9%)	8.84 ± 3.52	
Children	no	443 (42.2%)	8.21 ± 3.28	−2.98 (0.003)
	yes	607 (57.8%)	8.85 ± 3.56	
Occupation	managers, experts	171 (16.3%)	9.07 ± 3.65	1.13 (0.341)
	office workers	317 (30.2%)	8.47 ± 3.57	
	sales and service workers	130 (12.4%)	8.52 ± 3.07	
	skilled agricultural and fishery workers	4 (0.4%)	9.25 ± 4.03	
	technical workers, plant and machine operators and assemblers	34 (3.2%)	9.26 ± 3.48	
	simple labor workers	43 (4.1%)	8.79 ± 3.64	
	unemployed (housewife, student, etc.)	351 (33.4%)	8.37 ± 3.37	
Financial status	low ^a^	306 (29.1%)	7.94 ± 3.11	7.46 (0.001) a = b = c
	moderate ^b^	693 (66.0%)	8.84 ± 3.56	
	high ^c^	51 (4.9%)	8.82 ± 3.56	
Health status	low ^a^	110 (10.5%)	7.64 ± 2.96	9.29 (< 0.001) b,c > a
	moderate ^b^	723 (68.8%)	8.50 ± 3.42	
	high ^c^	217 (20.7%)	9.31 ± 3.70	
Experience of bioterrorism education	no	835 (79.5%)	8.07 ± 3.16	−8.76 (< 0.001)
	yes	215 (20.5%)	10.55 ± 3.83	

**Table 2:** The level of study variables (*N* = 1050).

Variables		Mean	SD	Min	Max	Range
Cognitive factors	Perceived probability	5.50	2.06	2.0	10.0	2–10
	Perceived seriousness	4.35	0.81	1.0	5.0	1–5
	Perceived personal impact	4.39	0.75	1.0	5.0	1–5
	Perceived coping efficacy	2.48	0.96	1.0	5.0	1–5
	Perceived resilience	2.46	0.53	0.3	3.9	0–4
Social–contextual factors	Perceived governmental preparedness	7.86	2.62	3.0	15.0	3–15
	Perceived front-line preparedness	10.46	3.18	4.0	20.0	4–20
Affective factors	Affective response to terrorism	3.42	0.98	1.0	5.0	1–5
	Anxiety	9.09	9.21	0.0	55.0	0–63
Bioterrorism preparedness		8.58	3.46	4.0	20.0	4–20

SD = Standard deviation.

**Table 3:** Correlations among study variables.

Variables		A	B	C	D	E	F	G	H	I	J
		Pearson’s r (*p*)									
Cognitive factors	A. perceived probability	1									
	B. perceived seriousness	0.13 (<0.001)	1								
	C. perceived personal impact	0.04 (0.237)	0.062 (<0.001)	1							
	D. perceived coping efficacy	0.05 (0.128)	−0.22 (<0.001)	−0.18 (<0.001)	1						
	E. perceived resilience	0.03 (0.289)	0.01 (0.751)	0.02 (0.489)	0.30 (<0.001)	1					
Social–contextual factors	F. perceived governmental preparedness	−0.03 (0.376)	−0.17 (<0.001)	−0.11 (<0.001)	0.40 (<0.001)	0.20 (<0.001)	1				
	G. perceived front-line preparedness	0.02 (0.523)	−0.17 (<0.001)	−0.13 (<0.001)	0.41 (<0.001)	0.26 (<0.001)	0.79 (<0.001)	1			
Affective factors	H. affective response to terrorism	0.42 (<0.001)	0.17 (<0.001)	0.20 (<0.001)	−0.07 (0.017)	0.05 (0.146)	−0.15 (<0.001)	−0.11 (<0.001)	1		
	I. anxiety	0.16 (<0.001)	0.01 (0.721)	0.02 (0.633)	−0.05 (0.103)	−0.24 (<0.001)	−0.07 (0.024)	−0.02 (0.546)	0.20 (<0.001)	1	
	J. bioterrorism preparedness	0.06 (0.054)	−0.16 (<0.001)	−0.14 (<0.001)	0.49 (<0.001)	0.39 (<0.001)	0.42 (<0.001)	0.47 (<0.001)	0.02 (0.548)	−0.01 (0.908)	1

**Table 4:** Factors influencing bioterrorism preparedness (*N* = 1050).

Variables	B	SE	β	t	*p*
(Constant)	−1.78	0.84		−2.14	0.033
Gender (ref = male)	−0.69	0.17	−0.10	−4.13	<0.001
Marital status (ref = no)	0.43	0.17	0.06	2.47	0.014
The experience of bioterrorism education (ref = no)	1.54	0.21	0.18	7.42	<0.001
Perceived personal impact	−0.24	0.11	−0.05	−2.10	0.036
Perceived coping efficacy	0.99	0.10	0.28	10.15	<0.001
Perceived resilience	1.24	0.17	0.19	7.31	<0.001
Perceived front-line preparedness	0.30	0.03	0.28	10.47	<0.001
R^2^ = 0.42, Adjusted R^2^ = 0.41 F = 106.68, *p* < *0*.001					

SE = Standard Errors.

## Data Availability

The data presented in this study are available on request from the corresponding author.
